# Graphene Oxide-Enhanced and Dynamically Crosslinked Bio-Elastomer for Poly(lactic acid) Modification

**DOI:** 10.3390/molecules29112539

**Published:** 2024-05-28

**Authors:** Bingnan Zhou, Cunai Zheng, Ruanquan Zhang, Shuyuan Xue, Botuo Zheng, Hang Shen, Yu Sheng, Huagui Zhang

**Affiliations:** 1 Fujian Province Key Laboratory of Polymer Science, College of Chemistry and Materials Science, Fujian Normal University, Fuzhou 350007, Chinashengyu@fjnu.edu.cn (Y.S.); 2 College of Materials and Chemical Engineering, Minjiang University, Fuzhou 350108, China

**Keywords:** poly(lactic acid), graphene oxide, epoxidized soybean oil, toughening

## Abstract

Being a bio-sourced and biodegradable polymer, polylactic acid (PLA) has been considered as one of the most promising substitutes for petroleum-based plastics. However, its wide application is greatly limited by its very poor ductility, which has driven PLA-toughening modifications to be a topic of increasing research interest in the past decade. Toughening enhancement is achieved often at the cost of a large sacrifice in strength, with the toughness–strength trade-off having remained as one of the main bottlenecks of PLA modification. In the present study, a bio-elastomeric material of epoxidized soybean oil (ESO) crosslinked with sebacic acid (SA) and enhanced by graphene oxide (GO) nanoparticles (NPs) was employed to toughen PLA with the purpose of simultaneously preserving strength and achieving additional functions. The even dispersion of GO NPs in ESO was aided by ultrasonication and guaranteed during the following ESO-SA crosslinking with GO participating in the carboxyl–epoxy reaction with both ESO and SA, resulting in a nanoparticle-enhanced and dynamically crosslinked elastomer (GESO) via a β-hydroxy ester. GESO was then melt-blended with PLA, with the interfacial reaction between ESO and PLA offering good compatibility. The blend morphology, and thermal and mechanical properties, etc., were evaluated and GESO was found to significantly toughen PLA while preserving its strength, with the GO loading optimized at ~0.67 wt%, which gave an elongation at break of ~274.5% and impact strength of ~10.2 kJ/m^2^, being 31 times and 2.5 times higher than pure PLA, respectively. Moreover, thanks to the presence of dynamic crosslinks and GO NPs, the PLA-GESO blends exhibited excellent shape memory effect and antistatic properties.

## 1. Introduction

In view of the fast consumption of non-renewable fossil fuels and the increasingly serious environment pollution caused by the extensive application of petroleum-based polymers, especially polyolefin plastics, developments in renewable-sourced and biodegradable polymers have been attracting great attention in the past decades [1,2,3,4]. Among the reported degradable polymers, poly(lactic acid) (PLA), readily synthesized from bio-mass, has been regarded as one of the most promising materials to substitute for conventional petroleum-based plastics thanks to its good strength and susceptibility to decompose via hydrolysis of ester groups. However, neat PLA is intrinsically brittle, exhibiting poor toughness with low elongation at break (<10%), which not only limits its application as a mechanical material but also makes it difficult to be processed by stretching [5,6]. In addition, to serve as a general plastic the high price of PLA needs to be reduced. Hence, how to toughen PLA at a low cost has become an important topic in the development of next-generation environment-friendly materials [7,8].

To tackle this problem, various PLA-toughening strategies have been developed in recent decades, with the most common one being physical or chemical blending with elastomers such as polyurethane, rubber, and polyesters [9,10,11]. The modifications can remarkably improve the toughness of PLA but at the sacrifice of sustainability, using petroleum-derived toughening agents, and with a significant loss of mechanical strength. Moreover, additional compatibilizers are usually required to improve the compatibility between PLA and polymeric tougheners, which complicates the blending process. Therefore, more and more studies are focusing on toughening PLA based on renewable resources, and better, on those with no need of compatibilizers. One type of toughener that has been extensively studied is vegetable oil and its derivatives. For instance, having flexible chains and an epoxide group which can react with the carboxylic acid or hydroxyl ends of PLA, epoxidized soybean oil (ESO) was recently employed by a few studies [12,13,14] to toughen PLA, being able to provide good compatibilization and an excellent toughening effect. Nevertheless, the significant strength sacrifice of blending with ESO still exists, even though ESO was crosslinked by diacids before blending, as reported in a few studies [15].

On the other hand, graphene oxide (GO) is an intriguing two-dimensional (2D) nanomaterial that has a thickness of ~0.34 nm [16] but has a high strength and elastic modulus [17,18], being about one hundred times as strong as steel. Moreover, there are multiple oxygen groups such as epoxy, carboxylic, and hydroxyl groups on the surface of GO, which are readily modifiable [19,20,21]. These make GO an excellent filler for polymers, including PLA, to improve their performance as well as to endow some new functions such as electronic and conductive properties. However, due to its strong tendency to aggregate, GO is difficult to disperse well within the PLA matrix, which calls for tedious modification of GO before blending to achieve its good dispersion in PLA, as reported in some earlier studies [22,23,24].

In this work, ESO was used to modify PLA as a bio-toughener in light of its good sustainability and the good compatibility guaranteed by the interfacial reaction. To mitigate the strength sacrifice, GO was ultrasonically introduced as a filler and bio-sourced sebacic acid (SA) was used as a dynamic crosslinker of ESO, as well as a regulator to facilitate GO dispersion, ultimately to enhance the performance of toughened PLA. The blend morphology, and thermal and mechanical properties, etc., were evaluated using scanning electron microscopy (SEM), differential scanning calorimetry (DSC), thermo-gravimetry (TG), as well as tensile and impact tests, etc.

## 2. Results and Discussion

### 2.1. Preparation and Characterization of GO-Filled ESO Elastomer (GESO)

**Figure 1:**
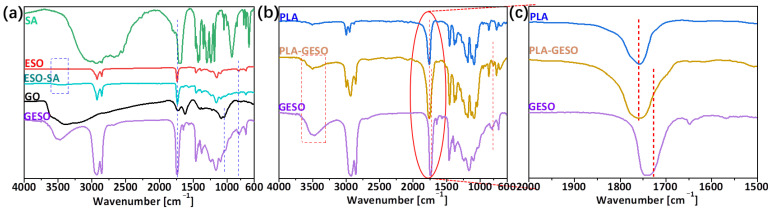
FTIR spectra of (**a**) SA-crosslinked ESO (ESO-SA) and GESO, with SA, ESO, and GO presented as references; (**b**) PLA/GESO blend, with PLA and GESO presented as references; (**c**) locally magnified section of (**b**). The dashed lines, and boxes are used for eye guide.

**Scheme 1:**
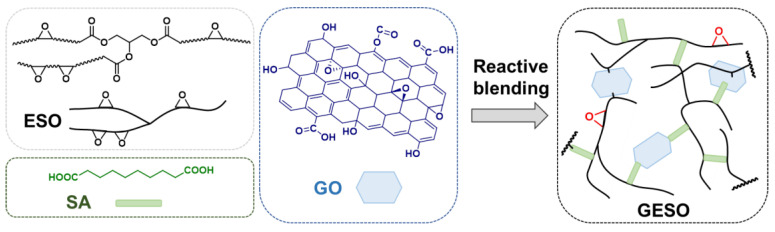
Expected GESO preparation via crosslinking reactions between ESO, SA, and GO via carboxyl groups and epoxy groups.

To better disperse GO in ESO, the mixture of ESO and GO was ultrasonicated at room temperature for 24 h to achieve a uniform dispersion before SA was added and stirred at 160 °C, whereby the ESO was covalently crosslinked by SA through a chemical reaction between the epoxy and carboxyl groups to form dynamic β-hydroxy ester moieties [25]. The reaction was confirmed from the FTIR spectra of SA-crosslinked ESO (named ESO-SA) and GESO (Figure 1). As compared to ESO, that has strong adsorption peaks at 902–843 cm^−1^, assigned to an epoxy group, the intensities of these peaks in ESO-SA were significantly reduced, verifying the consumption of epoxy groups by crosslinking reactions. Meanwhile, the appearance of a wide O-H absorption band around 3500 cm^−1^ in ESO-SA confirmed the generation of hydroxyl groups from the ring-opening reaction of epoxy groups into β-hydroxy ester moieties. This was also verified from the shift of the characteristic signal of the carbonyl C=O peak from acid type in SA, around 1700 cm^−1^, to ester type in ESO and GESO, peaking around 1738 cm^−1^. The presence of unreacted epoxy groups in ESO-SA is expected since the R value (carboxyl/epoxy group ratio) is 0.3. It is worth noting that during the crosslinking process the abundant epoxy and carboxyl groups in GO also participate in reactions with SA and ESO, making GO nanoparticles stably dispersed in the synthesized GESO (see Scheme 1). This was verified from the FTIR spectrum of GESO, where the characteristic adsorption band of GO around 1050 cm^−1^ and the enhanced O-H absorption band around 3500 cm^−1^ were observed. In addition, the signal of an epoxy group at 902–843 cm^−1^ remains strong in GESO, indicating the possibility of interfacial reaction with PLA during the melt blending.

**Figure 2:**
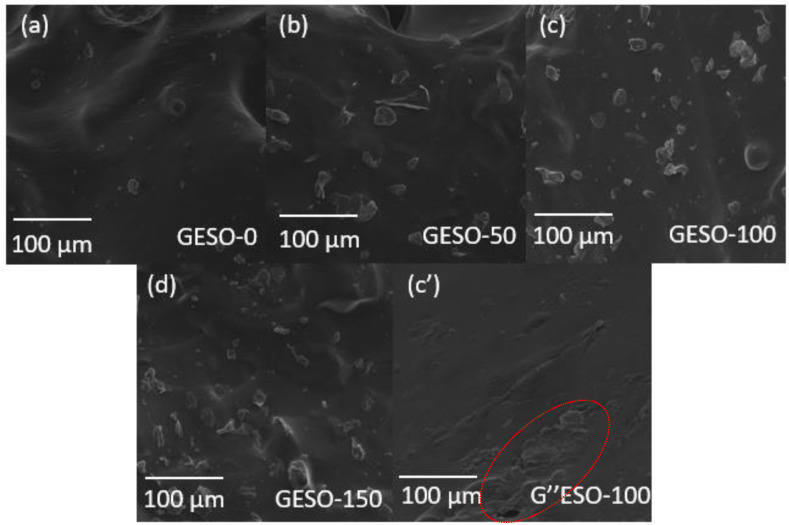
SEM images of GESO with different GO contents: (**a**) 0 mg, (**b**) 50 mg, (**c**) 100 mg, and (**d**) 150 mg; and (**c’**) the sample without ultrasonication during GO addition (100 mg).

Figure 2 shows the SEM micrographs of GESO with different GO contents, with the sample prepared without GO ultrasonication during its addition (i.e., G’’ESO) listed for comparison. As shown, the surface of GESO without GO is smooth and clean, while the surface of GESO with GO addition has obvious GO particles dispersed on the surface, and the particles on the surface become denser with the increase in GO content (Figure 2a–d). In contrast, in the sample without ultrasonication (Figure 2c’) evident GO particle agglomerations on the surface (red circle) were observed, indicating the importance of ultrasonication after GO addition in guaranteeing the uniform dispersion of GO in GESO.

**Figure 3:**
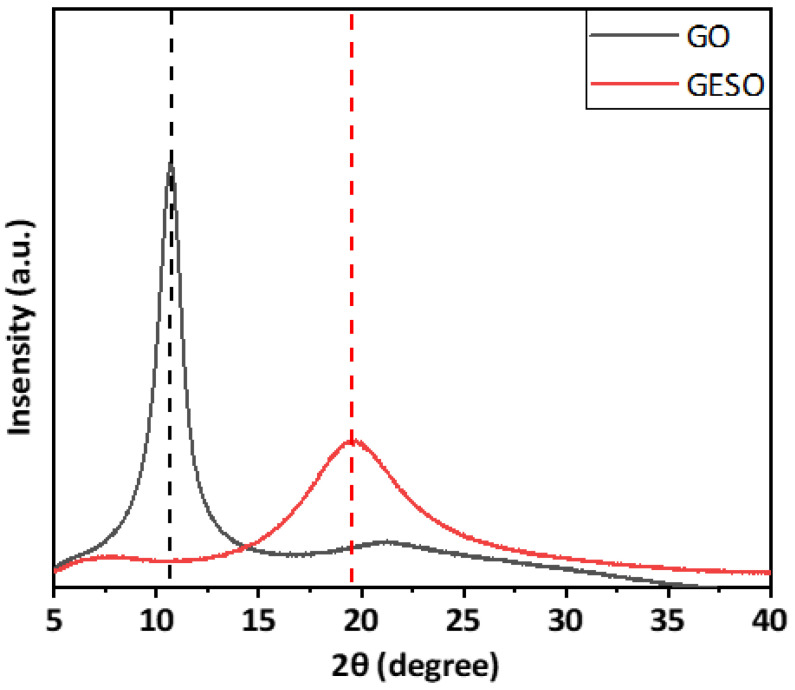
XRD profiles of GO and GESO.

Figure 3 shows the X-ray diffractograms of GO and GESO. In GO, an evident and sharp (001) diffraction peak is observed at 2θ around 10.9°, indicating a stacked structure with an interlayer distance of 0.811 nm according to Bragg’s equation [25]. In addition, the reflex observed at 2θ = around 21.0–22.0° is indicative of (002), caused by stacking between two or more graphene layers. However, in the XRD pattern of the GESO composite, no (001) reflex is observed, but a broad diffraction band around 2θ = 19.5° is indicative of an amorphous structure, which contains intercalated oxygen species between stacked multilayers of graphene.

The chemical composition of GO was further analyzed by XPS (Figure S2). In the wide-scan XPS survey spectra, significant emission peaks of C 1s and O 1s electrons can be observed at 286.9 eV and 534.6 eV, respectively. In the C 1s scan spectra, the C 1s peaks of GO can be deconvoluted to peaks at 284.8, 286.6, 286.9, and 288.8 eV, representing the carbon atoms in C-C, C-O, C=O, and C(O)C groups, respectively. This indicates the significant degree of oxidation in GO, with numerous oxygen-containing groups presented, most probably carboxyl, hydroxyl, and epoxy groups.

### 2.2. Preparation and Characterization of Toughened PLA-GESO Blends

**Scheme 2:**
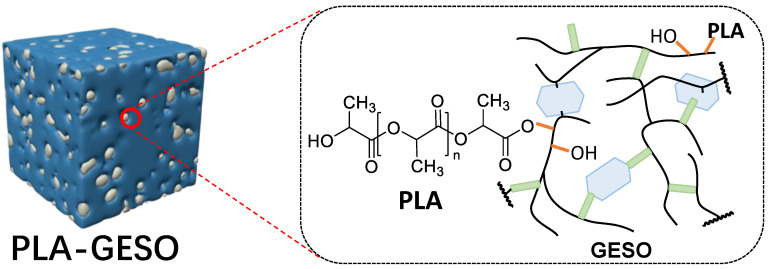
Expected morphology of PLA-GESO blend and the covalent bonding of PLA and GESO at the interface via ether bonds, resulting from reaction between epoxy groups in GESO and terminal carboxylic or hydroxyl groups in PLA chains.

The as-developed GESO was used to melt-blend with PLA, and the flexible triglyceride segments of soybean oil (SO) chains in GESO enable a good toughening effect on PLA. Moreover, the excess unreacted epoxy groups in GESO are supposed to react with the terminal hydroxyl and carboxylic groups of PLA by forming ether groups, as widely proposed in the literature [14,26], thus enhancing their phase compatibility (see Scheme 2). FTIR measurement was used to qualitatively prove the potential reaction. In the spectrum of the PLA-GESO blend (Figure 1b,c), in addition to the major peak of the carbonyl C=O at 1764 cm^−1^, corresponding to the alkyl ester group in PLA, a discernable shoulder around 1738 cm^−1^ was observed, corresponding to the C=O of β-hydroxy ester groups, which might be a result of the interfacial reaction between carboxyl acid ends in PLA and epoxy groups in GESO. This can also be verified by the significant O-H absorption band around 3500 cm^−1^ in the PLA-GESO blend as compared to neat PLA, as well as by the significantly reduced signal of the epoxy group (902–843 cm^−1^) as compared to that of GESO, which was consumed by the interfacial reaction. In the present study, PLA and GESO were blended at a mass ratio of 80/20, with a total mass of 60 g, and the GO content was varied in term of its loading in GESO (from 0 to 150 mg), with the blend denoted as P_80_E_20_R_0.3_G_x_ for short. In addition, the blends with different GO treatments, that is, with and without ultrasonication (denoted as P_80_E_20_R_0.3_G’’_x_) during their addition in ESO, and their addition during PLA/GESO blending (denoted as P_80_E_20_R_0.3_G’_x_), were compared to explore the effect of GO treatment, more exactly, GO dispersion.

#### SEM Characterization

**Figure 4:**
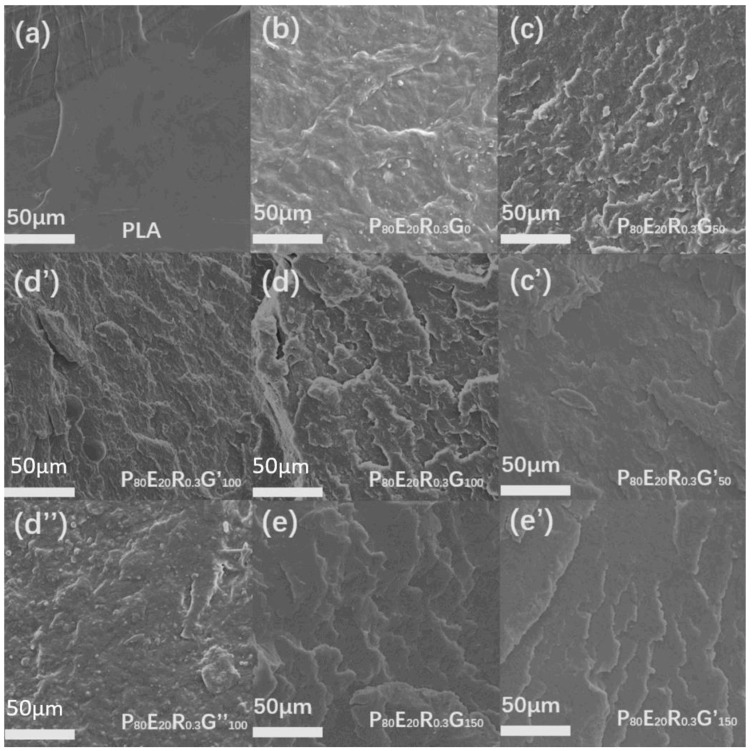
SEM micrographs of cryo-fractured surfaces of PLA-GESO mixtures containing different levels of GO and different modes of GO incorporation. (**a**) Pure PLA; (**b**) P_80_E_20_R_0.3_G_0_; (**c**) P_80_E_20_R_0.3_G_50_; (**d**) P_80_E_20_R_0.3_G_100_; (**e**) P_80_E_20_R_0.3_G_150_; (**c’**) P_80_E_20_R_0.3_G’_50_; (**d’**) P_80_E_20_R_0.3_G’_100_; (**e’**) P_80_E_20_R_0.3_G’_150_; and (**d’’**) P_80_E_20_R_0.3_G’’1_00_.

SEM was applied to characterize the morphology of the PLA-GESO blends. Figure 4 show SEM images of the low-temperature fracture surfaces of PLA-GESO blends (P_80_E_20_R_0.3_G_0_, P_80_E_20_R_0.3_G_50_, P_80_E_20_R_0.3_G_100_, P_80_E_20_R_0.3_G_150_, P_80_E_20_R_0.3_G’_50_, P_80_E_20_R_0.3_G’_100_, P_80_E_20_R_0.3_G’_150_ and P_80_E_20_R_0.3_G’’1_00_) with different GO contents added via different ways. Pure PLA was inspected as a reference. It is clear that pure PLA exhibits a typical brittle fracture morphology with a smooth fracture surface (Figure 4a). The surface of P_80_E_20_R_0.3_G_0_ has only a small number of protofibers and slight matrix deformation (Figure 4b), which suggests a tendency for ductile fracture. In contrast, the PLA-GESO samples containing GO show rougher fracture surfaces (Figure 4c–e’), indicating that the PLA matrix was deformed due to GESO plasticization during low-temperature fracture. The fracture surfaces of all the blend samples have quasi-laminar patterns but with no distinct phase boundaries observed (Figure 4c–e), thanks to the good compatibility, enhanced by interfacial reaction between ESO and PLA. When GO was ultrasonically dispersed in the blends, the fracture surfaces were rough and laminarly wrinkled, indicative of ductile fracture (Figure 4c–e). When GO was added to ESO without ultrasonication, the GO agglomerated in the synthesized GESO due to non-uniform dispersion (Figure S1a). Compared with the laminarly wrinkled surface in P_80_E_20_R_0.3_G_100_ (Figure 4d), where GO was ultrasonically dispersed, the surface of P_80_E_20_R_0.3_G’’1_00_ (Figure 4d’’), in which GO was not ultrasonicated, is much smoother and less wrinkled, indicating more brittle fracture. The surface of P_80_E_20_R_0.3_G’’1_00_ is much more similar to that of P_80_E_20_R_0.3_G_0_ (Figure 4b) in which GO is absent. The blends (P_80_E_20_R_0.3_G’_x_) prepared by adding GO during the polymer blending also formed more obvious rough structures (Figure 4c’–e’), but their rough, quasi-laminar, or wrinkled patterns were on larger scales than those of the corresponding ultrasonically dispersed blends. This suggests that both the presence of GO and the ultrasonic treatment when mixing GO with ESO play important roles in building a rougher structure of the composites for better performance.

### 2.3. Thermal and Crystallization Behaviors of PLA-GESO Blends

**Figure 5:**
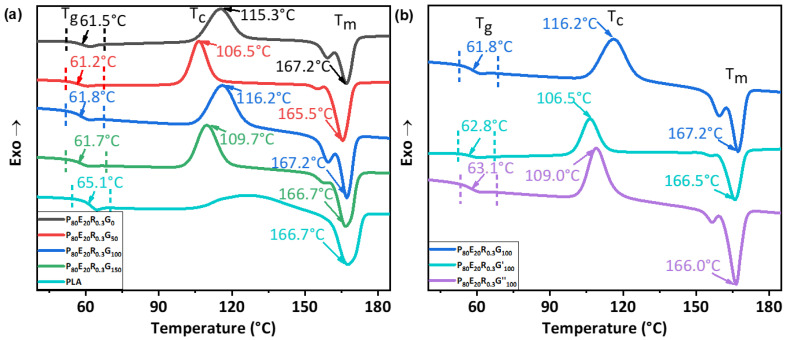
(**a**) DSC curves of PLA and PLA-GESO blends with different GO contents; (**b**) DSC curves of PLA-GESO blends with different GO treatments.

**Figure 6:**
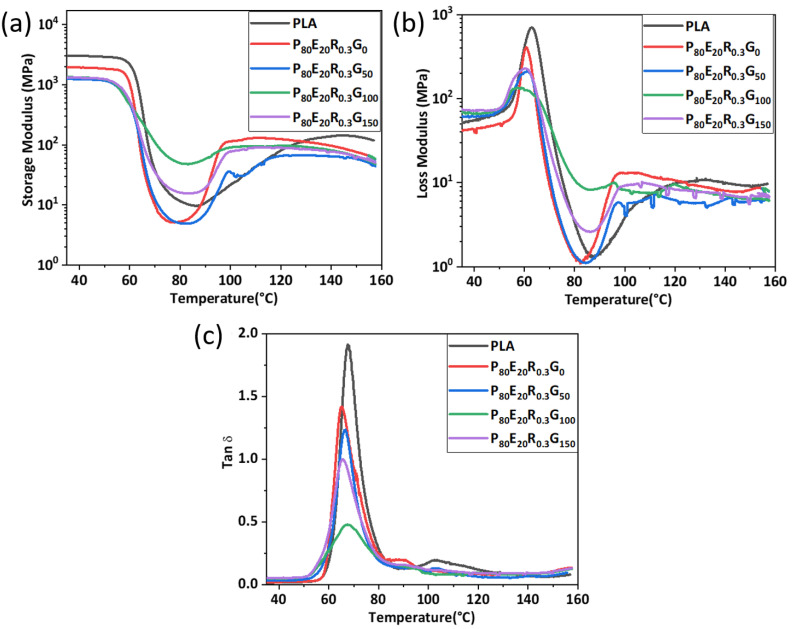
Temperature dependence of (**a**) storage modulus (E′), (**b**) loss modulus (E′′), and (**c**) damping factor (tan δ) for pure PLA and PLA-GESO blends with different GO contents.

Figure 5 shows the DSC curves of the pure PLA and PLA-GESO blends. The glass transition and melting characteristics of PLA crystals can be clearly observed in the DSC curves. The data for the glass transition temperature (T_g_), enthalpy of melting (Δ*H_m_*) (i.e., the integral of the melting peak), and the calculated crystallinity (*X_c_*) of the samples are summarized in Table 1. As shown in Figure 5, the addition of ESO reduced the T_g_ from 65.1 °C for pure PLA to 61.5 °C for the blends. This effect is also confirmed by the T_g_, determined by dynamic mechanical analysis (DMA), i.e., the temperature corresponding to the highest tan δ. The DMA results showed a decrease from 67.7 °C for pure PLA to 65.4–67.0 °C for PLA-GESO blends (i.e., P_80_E_20_R_0.3_G_0_, P_80_E_20_R_0.3_G_50_, P_80_E_20_R_0.3_G_100_, and P_80_E_20_R_0.3_G_150_) (Figure 6). In the blends with fixed PLA/GESO ratios, there is no significant change in T_g_ with increasing GO content but a slight alteration in the cold crystallization peak in the range between 100 and 120 °C. Figure 5b compares the DSC curves of the PLA-GESO blends prepared in different ways (GO sonicated, unsonicated, and GO added during blending). Compared to P_80_E_20_R_0.3_G_100_, P_80_E_20_R_0.3_G’_100_ (GO unsonicated) and P_80_E_20_R_0.3_G’’1_00_ (GO added directly during blending) have higher T_g_s and lower cold crystallization temperatures (T_c_) with smaller crystallization peaks, likely indicating that the non-uniformity of GO particles in the latter cases reduced phase compatibility whilst favoring cold crystallization, with the heterogeneity acting as a crystallization nucleating point.

As to the melting behaviors, the results showed that the PLA-GESO blends have similar melting temperatures (T_m_) to that of pure PLA (Table 1) but exhibit two melting peaks, with a minor peak detected at a lower temperature, likely indicating the presence of disordered α′-type crystals in addition to the ordered α-type crystals in PLA, or less perfect crystals versus more perfect crystals [27].

Moreover, as shown in Table 1, the crystallinity (*X_c_*) of the blends decreased slightly with the addition of GO-free GESO, but increased with the GO content in GESO. This is attributed to the fact that GO promotes the arrangement of polymer chains into ordered structures during the crystallization process, thus increasing the crystallinity of the polymers [28,29,30]. In addition, GO can serve as nuclei to promote the aggregation of polymers to form crystals, which further improves the crystallinity of PLA [31,32].

Furthermore, the dynamic mechanical properties of PLA-GESO blends were measured by temperature sweep tests, and the values of the storage modulus (E′), loss modulus (E″), and damping factor (tan δ ≡ E″/E′) versus temperature for PLA and PLA-GESO with different GO contents are depicted in Figure 6, while those for PLA-GESO with different GO treatments are depicted in Figure S3. The E′ curves of PLA-GESO blends showed similar trends to PLA, with E′ plateauing below 50 °C, and then, decreasing abruptly above this due to the glass transition from glassy to rubbery state, which corresponds to the large tan δ peak (Figure 6c). At higher temperatures, above 80 °C, small humps appear in both the E′ and E″ curves for all the samples, which are attributed to cold crystallization of PLA. The cold crystallization peak shown in the tan δ curves (Figure 6c) shifted to lower temperatures after the addition of GESO to PLA, suggesting that GESO promoted chain motions and rearrangements. This confirms the plasticizing effect of GESO on PLA, consistent with the DSC study.

Moreover, P_80_E_20_R_0.3_G_100_ has the smallest tan δ peak height, that is, the highest energy storage and lowest energy dissipation, which might suggest that the network structure of the P_80_E_20_R_0.3_G_100_ blend is superior to the other blends. In addition, the dynamical properties of PLA-GESO blends prepared with different GO treatments (P_80_E_20_R_0.3_G_100_ for GO sonicated, P_80_E_20_R_0.3_G’’1_00_ for GO unsonicated, and P_80_E_20_R_0.3_G’_100_ for GO added during blending) are compared (Figure S3), wherein P_80_E_20_R_0.3_G_100_ still has the smallest tan δ peak height, indicating the lowest damping behavior. That is, the sonication treatment of GO is of great significance for uniform dispersion to guarantee good performance.

**Figure 7:**
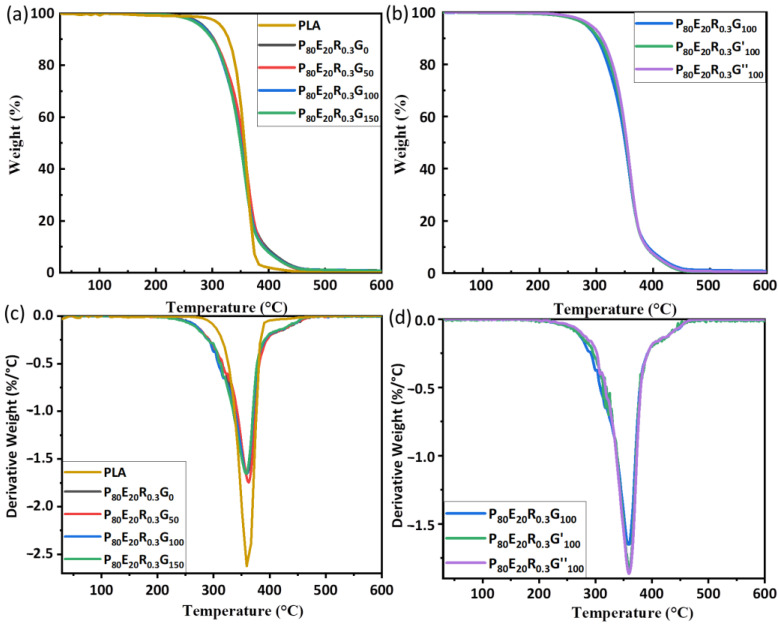
TGA curves (**a**,**b**) and derivative weight cures (**c**,**d**) of PLA and PLA-GESO blends with different GO contents(**a**,**c**); and PLA-GESO blends with different GO treatments(**b**,**d**).

TGA analysis was also conducted to study the effect of GESO on the thermal stability of the PLA blends, and the data are shown in Figure 7, with pure PLA presented as a control sample. Their temperatures at 10% weight loss (T_10_) and 50% weight loss (T_50_) are summarized in Table 2. As shown in Figure 7, the T_10_ of the PLA-GESO blends (303 °C for P_80_E_20_R_0.3_G_0_ and 302 °C for P_80_E_20_R_0.3_G_150_) is almost 30 °C lower than the T_10_ of pure PLA (328 °C). This is due to the presence of partially crosslinked ESO with a lower thermal stability than PLA, prone to decomposition. The T_10_ and T_50_ of the PLA-GESO samples with different amounts of GO are similar, which is due to the fact that the weight fraction of GO is low. More importantly, GESO enhanced the thermal stability of the blends at high temperatures, shifting the temperature of maximum decomposition rate (T_max_) from 375 °C in PLA to 383 °C (Table 2), and above that the decomposition of blends was slowed down with a much higher residual value than pure PLA. This is likely because some of the epoxy groups in GESO experienced ring-opening reactions with PLA, extending the molecular chain of PLA as well as forming crosslinking networks, and thus, improving the thermal stability. That is to say, the addition of GESO and especially its ring-open grafting to PLA plays a role in the construction of thermally stable toughened PLA blends. In addition, as shown in Figure 7b, the different GO treatments do not show much difference in their TGA data, confirming that the enhanced thermal stability in PLA-GESO blends mainly comes from the contribution of the interfacial interaction between GESO and PLA.

### 2.4. Mechanical Properties and Toughening Mechanism of PLA-GESO Blends

#### 2.4.1. Tensile Toughness Mechanism

**Figure 8:**
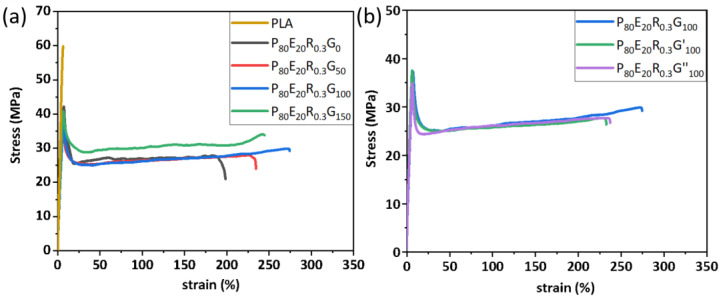
Tensile stress–strain curves of PLA and PLA-GESO. (**a**) PLA and PLA-GESO blends with different GO contents; (**b**) PLA-GESO blends with different GO treatments.

To evaluate the toughening effect of GESO on PLA, all the blend samples were subjected to tensile tests for tensile strength and toughness measurements, using pure PLA as a control sample. During stretching, all the PLA-GESO blends exhibited a pronounced stress whitening phenomenon, indicative of yielding and ductile fracture, completely different from the absence of stress whitening in pure PLA, that is known to have brittle fracture. Figure 8 show the stress–strain curves of the pure PLA and PLA-GESO blends with different GO contents (Figure 8a), and with different GO treatments (Figure 8b). As expected, pure PLA experienced a typical brittle damage upon stretching, with an ultimate tensile strength up to 60 MPa and a very low elongation at break (~6.5%), which is in agreement with earlier findings [33]. In contrast, the elongation at break of the PLA-GESO blends increased dramatically, up to 199.1% to 274.5%, with a subtle dependence on GO content (increase first and then decrease, peaking at P_80_E_20_R_0.3_G_100_), while the tensile strength still remained at a high level, in the range of 39 to 42 MPa. In addition, compared with P_80_E_20_R_0.3_G’_100_ and P_80_E_20_R_0.3_G’’1_00_, the elongation at break of P_80_E_20_R_0.3_G_100_ was significantly higher and the tensile strength was comparable or even higher, again verifying the importance of GO ultrasonication during its dispersion in ESO in achieving superior mechanical performance. In sum, PLA can be successfully toughened by blending with GESO, with an excellent tensile toughness and preserved strength, especially for P_80_E_20_R_0.3_G_100_, where GO was optimized at ~0.67 wt% (i.e., 100 mg in GESO). The better stress/strain dependence of P_80_E_20_R_0.3_G_100_ is probably due to the enhanced elasticity of GESO with increased GO loading as the GO can act as additional crosslinking points in GESO, while a too high GO loading (e.g., G_150_) might result in a relatively poor particle dispersion that negatively affects the dependence. This is also consistent with the DMA data that the P_80_E_20_R_0.3_G_100_ possess the lowest tan δ value (Figure 6c).

**Figure 9:**
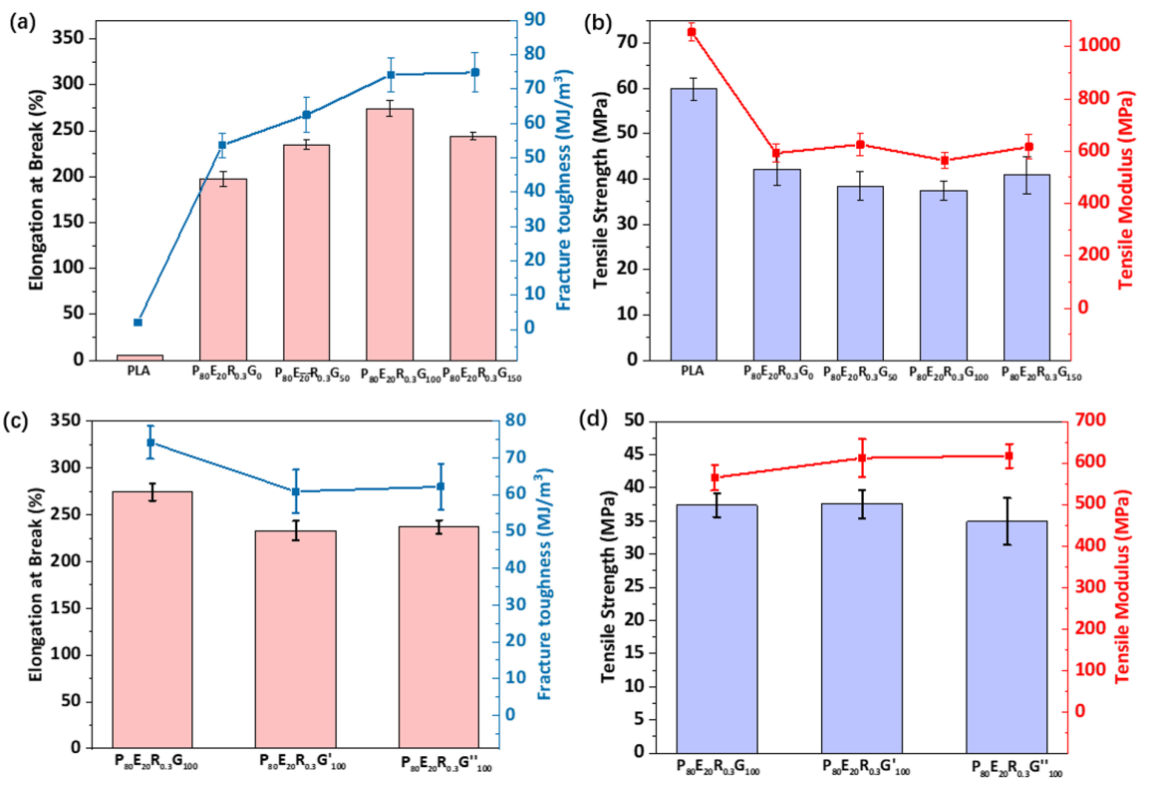
Tensile properties (tensile modulus, tensile strength, elongation at break, and fracture toughness) of PLA and PLA-GESO. (**a**,**b**) Pure PLA and PLA-GESO blends with different GO contents; (**c**,**d**) PLA-GESO blends with different GO treatments.

For better comparisons, Figure 9 summarizes the toughness data, including elongation at break and tensile toughness, calculated from the integral of the tensile curves, as well as the strength data, including tensile strength and tensile modulus, obtained from the stress–strain curves of the blends. As shown in Figure 9a, an increase in GO content significantly enhances the ductility of the PLA-GESO blends (PLA/GESO 8/2 *w*/*w*), with a significant increase in tensile toughness from 3.3 MJ/m^3^ for pure PLA to 53.6 MJ/m^3^ for P_80_E_20_R_0.3_G_0_, and this is further enhanced by GO to 62.5–74.8 MJ/m^3^ for P_80_E_20_R_0.3_G_50_ to P_80_E_20_R_0.3_G_150_. Among the samples, the elongation at break and tensile toughness of P_80_E_20_R_0.3_G_100_ are the highest, i.e., 274.5% and 74.8 MJ/m^3^, respectively. The tensile toughness reduction with further increase in GO is probably due to the increased difficulty of even dispersion of GO in ESO at high GO loading. On the other hand, the addition of SA-crosslinked ESO decreased the tensile strength and tensile modulus (Figure 9b) while toughening the PLA, but after the incorporation of GO the tensile toughness of PLA was further significantly enhanced (Figure 9a) without further loss of tensile strength (Figure 9b). This demonstrates that GO fillers play a role in enhancing the toughness while maintaining the strength of the material. Also confirmed from Figure 9c,d is the importance of GO sonication treatment.

**Figure 10:**
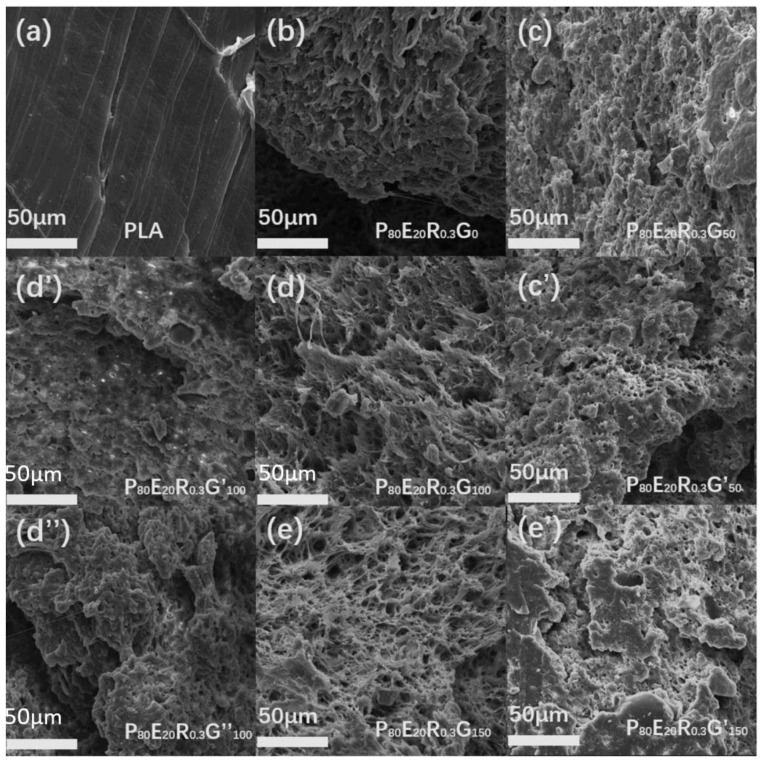
SEM images of the tensile fracture surfaces of PLA (**a**), P_80_E_20_R_0.3_G_0_ (**b**), P_80_E_20_R_0.3_G_50_ (**c**), P_80_E_20_R_0.3_G_100_ (**d**), P_80_E_20_R_0.3_G_150_ (**e**), P_80_E_20_R_0.3_G’_50_ (**c’**), P_80_E_20_R_0.3_G’_100_ (**d’**), P_80_E_20_R_0.3_G’_150_ (**e’**), and P_80_E_20_R_0.3_G’’1_00_ (**d’’**).

In order to gain a deeper understanding of the toughening mechanism of GESO on PLA under tensile deformation, the tensile fracture surfaces of PLA and PLA-GESO blends after tensile testing were observed by SEM. As shown in Figure 10, the fracture surface of PLA was smooth and flat (Figure 10a), which is a typical characteristic of brittle fracture; however, the fracture surfaces of all the PLA-GESO blends were rough. In particular, the highly oriented deformation of the PLA matrix with a threaded shape was widely observed in the blends (Figure 10b–e), which is evidence of a ductile fractured morphology originating from shear yielding of the matrix during stretching.

Significant differences in fracture surface morphology were observed between the blends with different GO contents, which exhibited very pronounced fracture elongation and tensile roughness. In the studied blends (Figure 10b–e), as compared to P_80_E_20_R_0.3_G_0_ the fracture surfaces of the GO-containing blends, e.g., P_80_E_20_R_0.3_G_100_ and P_80_E_20_R_0.3_G_150_, were much rougher, with a particularly high number of porous structures. The pores were likely caused by internal cavitation of the dispersed GESO elastomers during stretching, while some partial pull-out of GESO particles adhering to the fracture surface was also observed, indicating the possible occurrence of interfacial debonding cavitation. Cavities created during stretching are deemed to significantly dissipate tensile energy, leading to high elongation at break and tensile toughness [34,35]. Aside from the cavitations, continuous thinning of the matrix filaments occurred during stretching, which is also often considered to contribute to the toughening effect [36].

In addition, the comparison of P_80_E_20_R_0.3_G_50_, P_80_E_20_R_0.3_G_100_, and P_80_E_20_R_0.3_G_150_ with P_80_E_20_R_0.3_G’_50_, P_80_E_20_R_0.3_G’_100_, P_80_E_20_R_0.3_G’_150_, and P_80_E_20_R_0.3_G’_100_ reveals that the tensile roughness of the latter cases (Figure 10c’–e’,d’’) is much lower, and the orientation deformation is much smaller, with less PLA matrix filament observed. This proves that the treatment of GO with ultrasonication for better dispersion is superior to that without ultrasonication treatment and that the GO was added directly during melt blending. This is well verified by the high toughening behavior of P_80_E_20_R_0.3_G_100_, exhibiting an excellent elongation at break (274.5%) and tensile toughness (74.8 MJ/m^3^), while also maintaining the tensile strength at ~41 MPa (Figure 10). This demonstrates that the modification of ESO with GO as a nanofiller can enhance the toughness of PLA while maintaining its strength.

#### 2.4.2. Impact Toughening Mechanism

**Figure 11:**
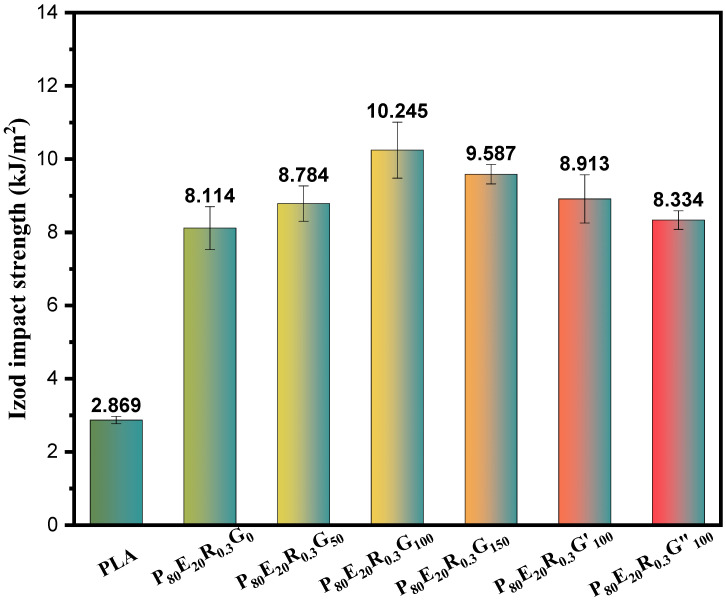
Notched Izod impact strength of PLA, P_80_E_20_R_0.3_G_0_, P_80_E_20_R_0.3_G_50_, P_80_E_20_R_0.3_G_100_, P_80_E_20_R_0.3_G_150_, P_80_E_20_R_0.3_G’_100_, and P_80_E_20_R_0.3_G’’_100._

Figure 11 shows the cantilever beam impact strength of the PLA and PLA-GESO blends with different compositions of GESO and different GO treatments. As widely reported in the literature [26], the cantilever beam impact strength of PLA has been measured to be as low as ~2.87 kJ/m^2^. After incorporation of 20 wt% GO-free GESO elastomer, the impact strength was significantly increased to 8.11 kJ/m^2^. Moreover, when GO was ultrasonically incorporated into GESO elastomers, the impact strength further increased, and then, decreased with GO content, peaking at P_80_E_20_R_0.3_G_100_ with a value of 10.25 kJ/m^2^, which is nearly 3.6 times higher than that of the original PLA, and 26.3% higher than that of P_80_E_20_R_0.3_G_0_ without GO. The enhancement is also greater than other GO treatment methods. Obviously, ESO and GO are effective components to improve the impact strength of PLA-GESO blends. Both the impact and tensile experiments show that the incorporation of GESO elastomers, especially when enhanced by GO nanofiller, can significantly improve the toughness of the PLA matrix, particularly optimized in P_80_E_20_R_0.3_G_100_ (i.e., 100 mg GO addition during GESO preparation) with an elongation at break of 274.5% (a fracture toughness of ~73 MJ/m^3^) and an impact strength of 10.25 kJ/m^2^, as compared to that of 6.5% (~2 MJ/m^3^) and 2.87 kJ/m^2^ for neat PLA.

**Figure 12:**
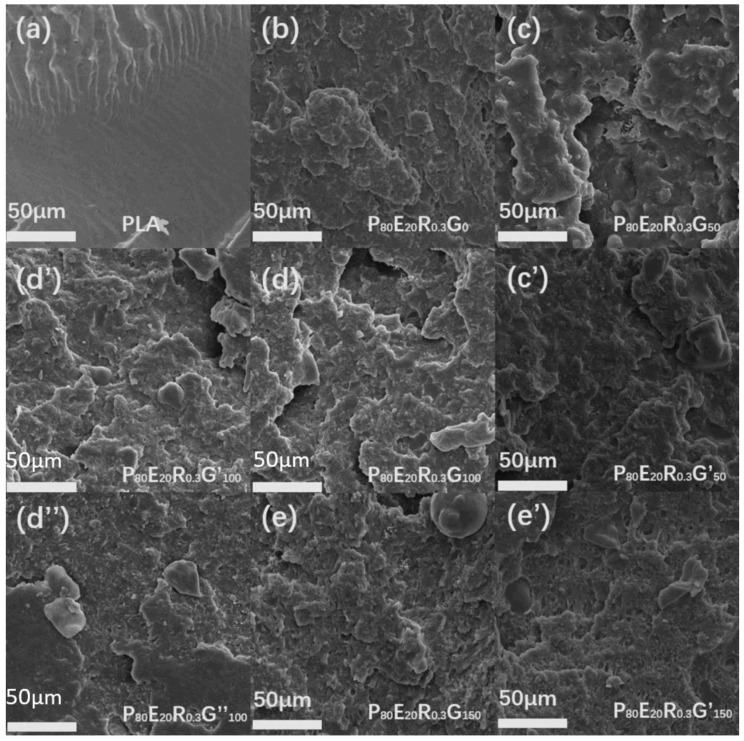
SEM images of impact fracture surfaces of PLA (**a**), P_80_E_20_R_0.3_G_0_ (**b**), P_80_E_20_R_0.3_G_50_ (**c**), P_80_E_20_R_0.3_G_100_ (**d**), P_80_E_20_R_0.3_G_150_ (**e**), P_80_E_20_R_0.3_G’_50_ (**c’**), P_80_E_20_R_0.3_G’_100_ (**d’**), P_80_E_20_R_0.3_G’_150_ (**e’**), and P_80_E_20_R_0.3_G’’1_00_ (**d’’**).

Figure 12 shows SEM images of notched impact fracture surfaces of PLA-GESO blends with different GO contents and different GO treatments. Due to brittle fracture, pure PLA exhibits a highly smooth impact fracture surface (Figure 12a). In contrast, all the PLA-GESO samples have very rough surfaces. In particular, the laminated and wrinkled structures of the PLA matrix with embedded GESO elastomer and large cavities generated from impact deformation are clearly observed (Figure 12b–e). The presence of such a rough surface and extensive plastic deformation of the PLA matrix clearly indicates the presence of ductile fracture in PLA-GESO during the impact experiments and the realization of a high cantilever beam notched impact strength (Figure 11).

### 2.5. Shape Memory Properties of PLA-GESO Blends

**Figure 13:**
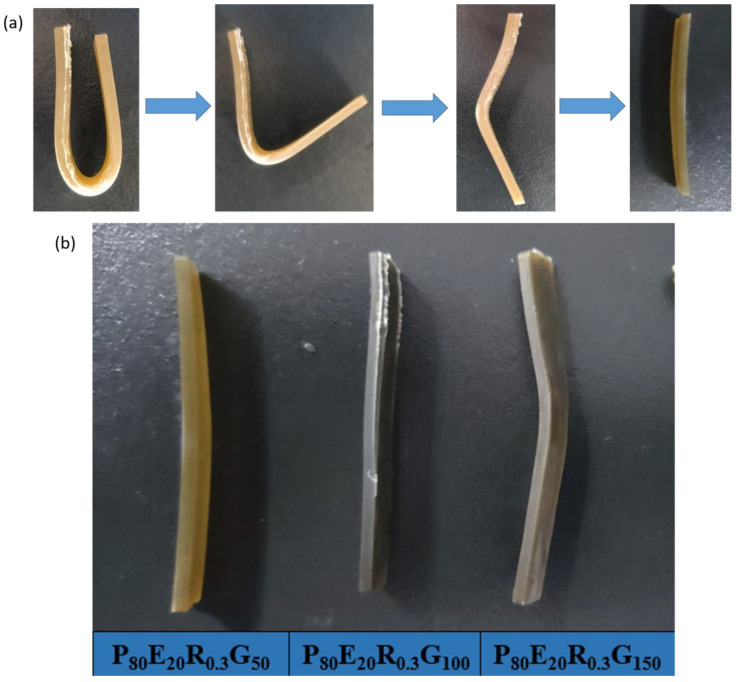
(**a**) Heated shape recovery process of P_80_E_20_R_0.3_G_50_ from a bent “U” shape fixed at room temperature. (**b**) Final state of P_80_E_20_R_0.3_G_50_, P_80_E_20_R_0.3_G_100_, and P_80_E_20_R_0.3_G_150_ samples after shape recovery.

As a typical thermoplastic polymer, PLA was reported to have a thermally responsive shape memory effect, finding applications in some specific fields [37]. However, the blending with tougheners might affect its shape memory performance. In the current work, thanks to the presence of adaptable covalent β-hydroxy ester moieties formed through chemical reactions between the epoxy and carboxyl groups between ESO and SA, as well as between them and GO, the dynamic crosslinked PLA-GESO blends were expected to show shape memory performance, with few effects from the addition of GESO. For this, a folding–unfolding shape memory test was performed (Figure S4), with a rectangular specimen bent into a “U” shape above its T_g_ (80 °C used) and cooled to ambient temperature to fix the shape, then heated again above 80 °C to see its shape recovery behavior. Figure 13a shows an example of the shape recovery process of the bent P_80_E_20_R_0.3_G_50_ sample upon heating. At the onset of shape recovery, the shape recovery rate increased slowly as the temperature increased. As the temperature was further increased, all the blend specimens showed a monotonous and sharp increase in shape recovery before reaching a stable state. As shown in Figure 13b, when reaching 80 °C, the shape recovery of the P_80_E_20_R_0.3_G_50_, P_80_E_20_R_0.3_G_100_, and P_80_E_20_R_0.3_G_150_ specimens was 97.2%, 97.1%, and 92.5%, respectively. This demonstrates a good shape recovery property of the PLA-GESO blends as expected, albeit an increased addition of GO, particularly exceeding G_100_, slightly decreases the recovery degree, which might be due to the relatively poor dispersion of GO nanoparticles that affects the polymer chain mobility.

### 2.6. Conductive Properties of PLA-GESO Blends

**Figure 14:**
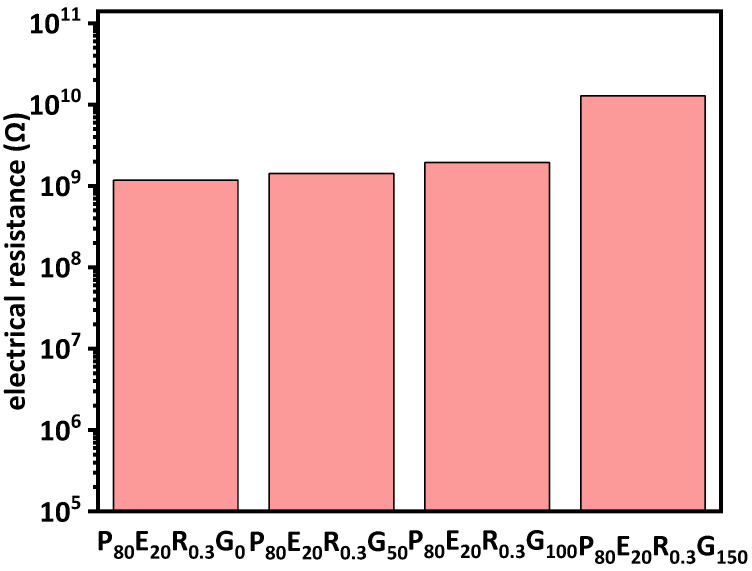
The electrical resistance of PLA-GESO blends with different GO loadings.

Figure 14 shows the electrical resistance values of the PLA-GESO blends with varied GO loadings. It was reported that the material with a resistance value greater than 10^12^ Ω can be considered as an electrical insulator that is prone to generating accumulated charge on the surface and cannot be discharged. However, for the values lying in between 10^6^ Ω and 10^11^ Ω the material can be considered as an antistatic material that accumulates less charge and the charge is easier to discharge [38,39]. As shown in Figure 14, the PLA-GESO blends have electrical resistances between 10^9^ Ω and 10^10^ Ω, exhibiting certain conductive and antistatic properties, which can also be confirmed by the glossy surface with little dust adhered after storage for one week in an ambient environment (Figure S5). Note that the presence of certain conductive properties of PLA-GESO blends is probably due to the abundance of electron-rich groups (e.g., the carboxyl and hydroxyl groups, etc.) in ESO, and a low amount of GO loading negligibly affects the performance except for P_80_E_20_R_0.3_G_150_ that has a higher resistance. The higher resistance might be due to the consumption of the electron-rich groups by GO and the relatively poor particle dispersion at such a high loading, which, however, is not enough to form interconnected networks.

## 3. Materials and Methods

### 3.1. Materials

Epoxidized soybean oil (ESO, AR) and sebacic acid (SA, AR) were purchased from Chengdu McCarthy Chemical Co. Graphene oxide (GO, water dispersion), 4-dimethylaminopyridine (DMAP, AR), and polylactic acid (PLA) were purchased from Hangzhou Gaoxi Technology Co., Ltd., Hangzhou, China, Shanghai Titan Technology Co., Ltd., Shanghai, China, and NatureWorks, Minneapolis, MN, USA, respectively. All the reagents were used as received.

### 3.2. Preparation Methods

#### 3.2.1. Dispersion of Graphene Oxide in Epoxidized Soybean Oil

First, the commercial GO dispersion was lyophilized to obtain GO powders by a freeze-dryer. Then, 50 mg, 100 mg, 150 mg, and 200 mg of GO were added to 15 g of epoxidized soybean oil (ESO), and homogenized using an ultrasonic sonication bath for 24 h, thereby GO was uniformly dispersed in the ESO. Dispersion of GO in ESO instead of PLA is to avoid particle aggregations because during the post-crosslinking process of ESO with SA the GO would participate in the reactions that facilitate its even dispersion. For clarity, the sample coding (i.e., P_80_E_20_R_0.3_G_x_) also refers to the GO quantity loaded in ESO instead of PLA as well (see below).

#### 3.2.2. Preparation of GO-Grafted Crosslinked ESO (GESO)

Vulcanization of ESO with sebacic acid (SA) was carried out at 160 °C using 4-dimethylaminopyridine (DMAP) as a catalyst. The feed weights of SA and ESO were determined based on a stoichiometric ratio (R = carboxyl/epoxy group) of 0.3. The catalyst DMAP content was 0.5 wt% of ESO. According to the formula, 1.9 g of SA and 75 mg of DMAP were added to a beaker of the GO-ESO dispersion made in the previous step. Then, the mixture was heated at 160 °C under magnetic stirring with protection of a nitrogen atmosphere to induce vulcanization until a black gel was formed. The formed ESO gel, consisting of GO and SA, is referred as GESO, and its appearance is shown in Figure S1. For comparison, a control sample was prepared without ultrasonication during GO dispersion in ESO but further reacted with SA under identical conditions. The product is referred to as GO-ESO, with the appearance shown in Figure S1a.

#### 3.2.3. PLA Blending with GESO

The blending of polylactic acid (PLA) with GESO was carried out in an HAAKE two-rolls mixer torque rheometer (PheoDrive 4, Karlsruhe, Germany). Before processing, PLA was vacuum-dried at 80 °C for 12 h. In a typical procedure, as the temperature in the torque rheometer reached 180 °C, the rotor speed of the rolls was set to 80 rpm, then 48 g of PLA and 12 g of GESO (weight ratio 80/20) were added, and the blending was maintained till the torque started to decrease after a plateau (~12 min), after which milling was stopped and the sample was discharged from chamber. The as-obtained PLA/GESO blend was abbreviated as P_80_E_20_R_0.3_G_x_ (the subscripts P and E denote weight fractions of PLA and ESO elastomer, respectively; the subscript R denotes the ratio of the carboxyl groups of SA to epoxy groups in ESO; and Gx denotes the mass of GO added to GESO in mg). For comparison, a blend with GO added during the blending process of PLA with SA-crosslinked ESO instead of pre-mixing GO in ESO was prepared and referred to as P_80_E_20_R_0.3_G’_x_, and the PLA/GESO blend prepared based on the GO addition to ESO without ultrasonication was referred as P_80_E_20_R_0.3_G’’x. Likewise, neat PLA was processed under the same conditions to prepare a control sample.

Subsequently, the obtained blend samples were crushed into granules and injection-molded at 180 °C and 0.5 MPa on an injection molding machine (SZS-20, Wuhan Ruiming, Wuhan, China) (mold temperature fixed at 40 °C, injection time ~2 s, molding time ~8 s) into standard bar specimens according to the standard of American Society for Testing and Materials (ASTM) D638 [40] for subsequent mechanical tests (i.e., tensile and impact tests).

### 3.3. Characterizations

#### 3.3.1. Fourier Transform Infrared (FTIR)

FTIR spectra of ESO and SA-crosslinked ESO (ESO-SA) samples were collected in a Thermo Nicolet 5700 FTIR spectrometer with a scanning range of 600 to 4000 cm^−1^ and a minimum of 36 scans using the KBr pellet method. The dried sample was ground into powder and diluted in KBr powder by about 100 times in weight and pressed into a pellet before measurement. The samples are not known to have any interaction with KBr that may affect the measurement.

#### 3.3.2. Scanning Electron Microscopy (SEM)

The sample morphology was characterized using a cold field emission scanning electron microscope (SEM, Regulus 8100, HITACHI, Hitachi, Japan) operated at a current of 10 μA and an accelerating voltage of 5.0 kV. The samples were sprayed with gold prior to the analysis.

#### 3.3.3. X-ray Diffraction (XRD)

Powder XRD patterns of the GO and GESO samples were measured using an X-ray powder diffractometer (X’Pert PXRD from Panalytical, Almelo, The Netherlands) with a copper target (1.54 Å) radiation source. The instrument was operated at 40 kV and 40 mA with a scanning angle (2θ) ranging from 5° to 40°.

#### 3.3.4. X-ray Photoelectron Spectroscopy (XPS)

The XPS data (full survey and high-resolution scan) of the neat GO sample were collected using an ESCALAB Xi+ (Thermo Fisher Scientific, Waltham, MA, USA) with Al Kα radiation (1486.6 eV).

#### 3.3.5. Thermogravimetric Analysis (TG)

A thermogravimetric analyzer (TGA2 from Mettler-Toledo, Switzerland) was used to characterize the thermal stability of samples in term of weight loss with temperature. The measurement was performed with the temperature increased from 0 °C to 600 °C at a heating rate of 10 °C/min using nitrogen as a protective atmosphere at a flow rate of 50 mL/min.

#### 3.3.6. Differential Scanning Calorimeter (DSC)

The thermal behaviors of samples were characterized using a differential scanning calorimeter (DSC3 STAR from Mettler-Toledo, Greisensee, Switzerland). A 10 ± 0.2 mg sample was encapsulated in an aluminum pot. Under a nitrogen flow at a rate of 50 mL/min, the sample was heated from 30 °C to 200 °C at a heating rate of 10 °C/min, followed by cooling to −50 °C at a cooling rate of −10 °C/min, and reheating to 200 °C at a heating rate of 10 °C/min. The DSC curves from the second heating cycle were used for analysis. The crystallinity of the PLA matrix (*X_c_*) in the blends was estimated using the following Equation (1):

(1) $$X_{C}=\frac{∆H_{m}}{w_{f}∆H_{m}^{{}^{\circ}}}\times 100$$

where ∆*H_m_* is the melt enthalpy of the analyzed sample and *w_f_* is the mass fraction of PLA in the blend. $∆H_{m}^{{}^{\circ}}$ is the melt enthalpy of completely crystalline PLA, i.e., 93.7 J/g.

#### 3.3.7. Tensile Test

In accordance with the Chinese Standard GB/T1040.1-2006 [41], the mechanical properties of the PLA-GESO blends, including tensile strength and elongation at break, were tested using a universal testing machine (LR5K PLUS, from LLOYD Instruments,Hampshire, UK) with a maximum tensile force of 500 N. A tensile experiment was carried out at room temperature (25 ± 2 °C) with a tensile rate of 10 mm/min. Each sample was tested five times using specimens prepared in the same batch, and the average value was used as the result for analysis.

#### 3.3.8. Impact Strength Test

In accordance with the Chinese Standard GB/1043-2008 [41], the impact strength of the PLA/GESO blend specimens was measured using a 50 N simply supported beam impact tester (ZBC1251 from SANS, Shenzhen, China). Each sample was tested five times at an ambient temperature of 25 ± 2 °C, and the average value was used as the result for analysis.

#### 3.3.9. Dynamic Mechanical Thermal Analysis (DMA)

The dynamic mechanical properties of the samples were measured using a Q800 dynamic mechanical thermal analyzer (TA instrument, New Castle, DE, USA). The samples were tested using a single cantilever clamp in a programmed temperature mode at a frequency of 1 Hz and an oscillation amplitude of 15 μm. The sample (size 10.0 × 30.0 × 2.0 mm) was heated from 35 °C to 160 °C at a heating rate of 3 °C/min. The deformation strain was set to 0.02% in the linear viscoelastic regime of the specimen. The storage modulus (E′), loss modulus (E″), and loss factor (tan δ ≡ E″/E′) were recorded and plotted against temperature.

#### 3.3.10. Shape Memory Performance Test

The shape memory performance of the blend samples was simply evaluated via a “folding–unfolding shape memory test”, as illustrated in Figure S4. In particular, the sample was first heated at 80 °C, above the glass transition temperature of PLA (~65 °C), and a certain external force was applied to bend the sample to reach the maximum deformation angle θ_max_, ~180°, forming a “U” shape, which was held for a certain period of time. Then, the sample was quickly cooled to room temperature with the force applied continuously for 2 min, maintaining the shape of the bent sample. After that, the force was released and the sample slightly bounced back, and the deformation angle of the sample was recorded as θ_fixed_. Lastly, the sample was reheated to the heat deflection temperature (80 °C) at a rate of 5 °C/min. The angular change θ_i_ of the sample as the temperature increased was recorded and the final bending angle was denoted as θ_final_. The angular change in the sample over time was also recorded.

#### 3.3.11. Conductive Performance Test

PLA/GESO blends with different amounts of GO were processed under identical conditions into specimens with the same dimensions. The electrical resistance was measured using an electrochemical workstation (CHI604E, Chenhua, Shanghai, China), based on a sine wave AC power source with a constant voltage of 1 V. Measurement was conducted with the two ends of the electrode connected to the ends of a rectangular-shaped sample with a length of 1 cm. Moreover, the samples were stored in the laboratory under the same conditions for one week and the dust absorption situation on the sample surface was evaluated by naked eye.

## 4. Conclusions

In the present study, graphene oxide (GO) nanoparticles (NPs) were dispersed with the aid of ultrasonication in epoxidized soybean oil (ESO), which was then crosslinked with sebacic acid (SA) to prepare a nanoparticle-enhanced dynamically crosslinked elastomer (GESO). The crosslinking was achieved via the carboxyl–epoxy reaction between ESO and SA, whilst GO was found to participate in the reactions.

Subsequently, the GESO was reactively blended with PLA as a toughener and the good compatibility guaranteed via interfacial reaction between PLA and GESO was confirmed by FTIR as well as by the T_g_ reduction observed in DSC. For comparison, PLA-GESO blends with different GO treatments, including GO unsonicated and GO added during the blending process, were studied as control samples. The GESO was demonstrated to favorably toughen PLA with the strength simultaneously preserved, and the GO content was found to be a crucial factor. Likewise, ultra-sonication treatment was demonstrated to be necessary to achieve good performance. The best mechanical properties were obtained in P_80_E_20_R_0.3_G_100_, with a GO loading optimized at ~0.67 wt% (100 mg in GESO), giving an elongation at break of ~274.5% and an impact strength of ~10.2 kJ/m^2^, which are 31 times and 2.5 times higher than pure PLA, respectively. The tensile strength of ~42 MPa was maintained. The excellent toughening effect was verified to be dominated by the internal cavitation of the GESO phase as well as the PLA matrix yielding, as confirmed by SEM observations of a rough surface, cavity pores, and thinning filaments. Moreover, the PLA-GESO blend was also demonstrated to have a shape memory performance and a good antistatic property thanks to the presence of dynamic crosslinks and functional GO nanoparticles. In a word, a GO-enhanced dynamically crosslinked ESO bio-elastomer allows modification of PLA with a trade-off on toughness and strength, providing a new avenue towards developing new high-performance and functional renewable plastics.

## Figures and Tables

**Table 1:** Thermal properties of pure PLA and PLA-GESO blends determined from DSC tests.

Sample	T_g_ (°C)	Δ*H_m_* (J/g)	Δ*H_c_* (J/g)	*X_c_* (%)
PLA	65.1	39.7		42.4
P_80_E_20_R_0.3_G_0_	61.5	29.5	28.3	39.3
P_80_E_20_R_0.3_G_50_	61.2	34.6	32.5	46.1
P_80_E_20_R_0.3_G_100_	61.8	40.8	39.4	54.4
P_80_E_20_R_0.3_G_150_	61.7	40.8	38.6	54.4
P_80_E_20_R_0.3_G’_100_	62.8	29.6	28.5	39.4
P_80_E_20_R_0.3_G’’1_00_	63.1	40.5	39.3	54.0

**Table 2:** T_10_, T_50_, and T_max_ obtained from TGA curves of PLA and PLA-GESO blends.

Sample	T_10_ (°C)	T_50_ (°C)	T_max_ (°C)
PLA	328	357	375
P_80_E_20_R_0.3_G_0_	303	352	382
P_80_E_20_R_0.3_G_50_	302	354	383
P_80_E_20_R_0.3_G_100_	302	352	383
P_80_E_20_R_0.3_G_150_	302	351	383
P_80_E_20_R_0.3_G’_100_	202	352	383
P_80_E_20_R_0.3_G’’1_00_	303	353	383

## Data Availability

Data are contained within the article.

## Supplementary Materials

The following supporting information can be downloaded at: https://www.mdpi.com/article/10.3390/molecules29112539/s1, Figure S1: GESO elastomeric gels prepared from blend crosslinking of ESO and GO by SA: (a) GO was added to ESO without ultrasonication; (b) GO was added to ESO with ultrasonication for even dispersion; Figure S2: (a) XPS wide-scan and high-resolution (b) carbon scan spectra of GO; Figure S3: Temperature dependence of (a) storage modulus (E′), (b) loss modulus (E″), and (c) damping factor (tan δ) of PLA-GESO blends obtained with different GO treatments; Figure S4: Schematic diagram of the shape memory performance test; Figure S5: Pictures for anti-electrostatic test of P_80_E_20_R_0.3_G_0_, P_80_E_20_R_0.3_G_50_, P_80_E_20_R_0.3_G_100_, and P_80_E_20_R_0.3_G_150_ samples stored after one week in the ambient environment.
