# Supplementary material for: Graphene Oxide-Enhanced and Dynamically Crosslinked Bio-Elastomer for Poly(lactic acid) Modification
Source: Molecules. 2024 May 28;29(11):2539. doi: 10.3390/molecules29112539 (PMC11173449; doi:10.3390/molecules29112539)
Supplement: Supplementary file 1 [file molecules-29-02539-s001.zip › molecules-3010499-supplementary.pdf]

Supplementary Materials

# Graphene Oxide Enhanced and Dynamically Crosslinked Bio-elastomer for Poly(lactic acid) Modification

Bingnan Zhou[1, †], Cunai Zheng[1, †], Ruanquan Zhang[1], Shuyuan Xue[1], Botuo Zheng[1], Hang Shen[2,*], Yu Sheng[1], Huagui Zhang[1,*]

[1]College of Chemistry and Materials Science, Fujian Province Key Laboratory of Polymer Science, Fujian Normal University, Fuzhou, 350007, China

[1]College of Materials and Chemical Engineering, Minjiang University, Fuzhou 350108, China

* Correspondence: hang.shen@outlook.com (H.S.); huagui.zhang@fjnu.edu.cn (H.Z.)

† These authors contributed equally to this work.

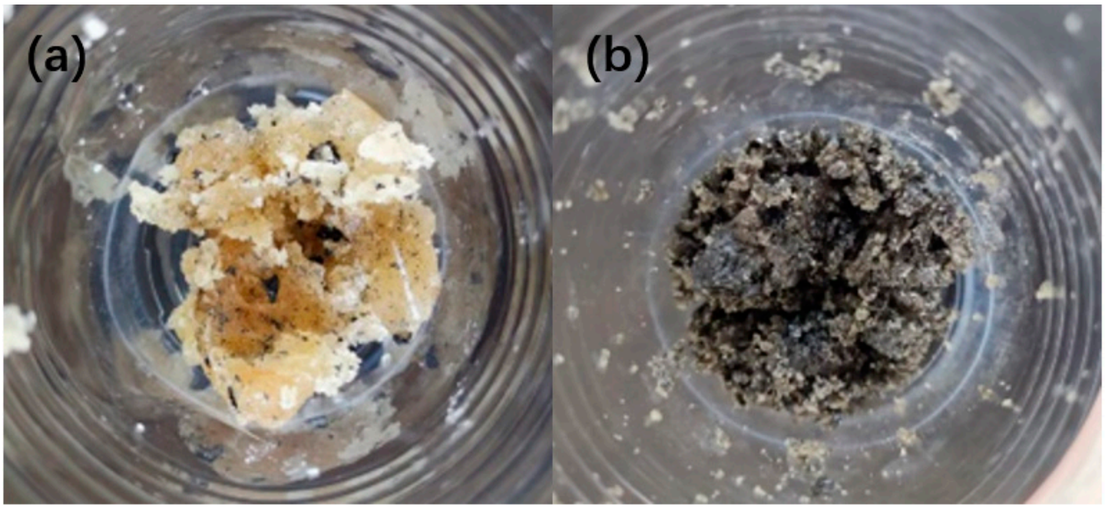


Figure S1. GESO elastomeric gels prepared from blend crosslinking of ESO and GO by SA: (a) GO was added to ESO without ultrasonication; (b) GO was added to ESO with ultrasonication for even dispersion

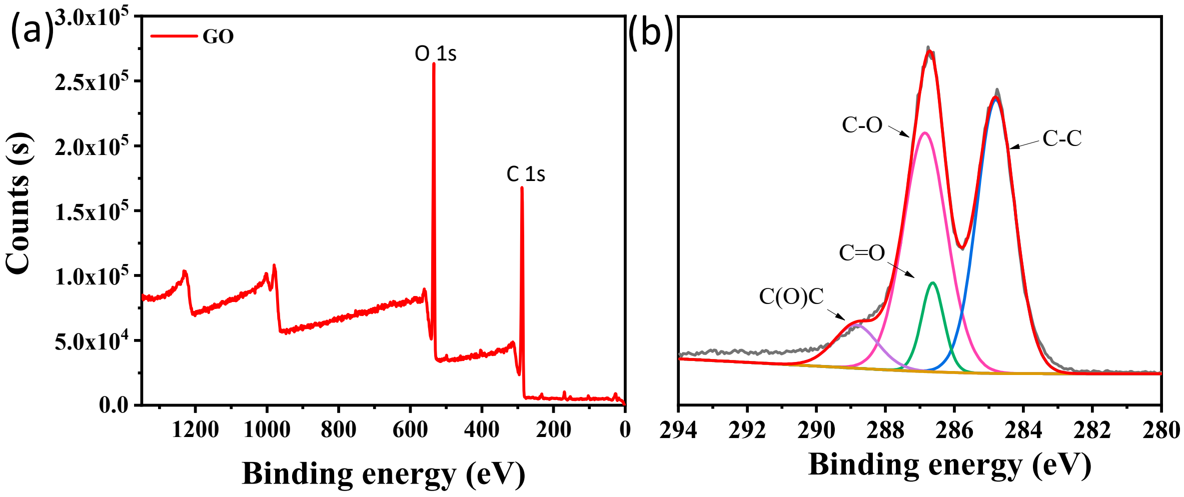


Figure S2. (a) XPS wide-scan and high-resolution (b) carbon scan spectra of GO.

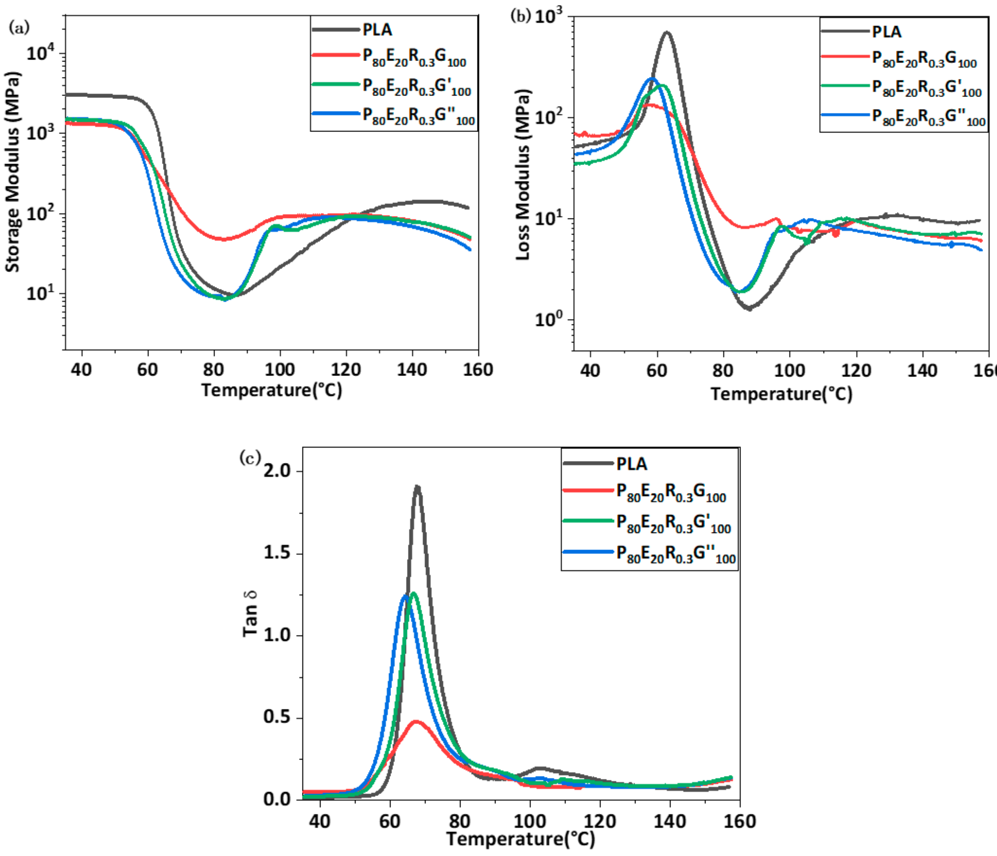


Figure S3. Temperature dependence of (a) storage modulus (E'), (b) loss modulus (E'') and (c) damping factor (tan δ) of PLA-GESO blends obtained with different GO treatments

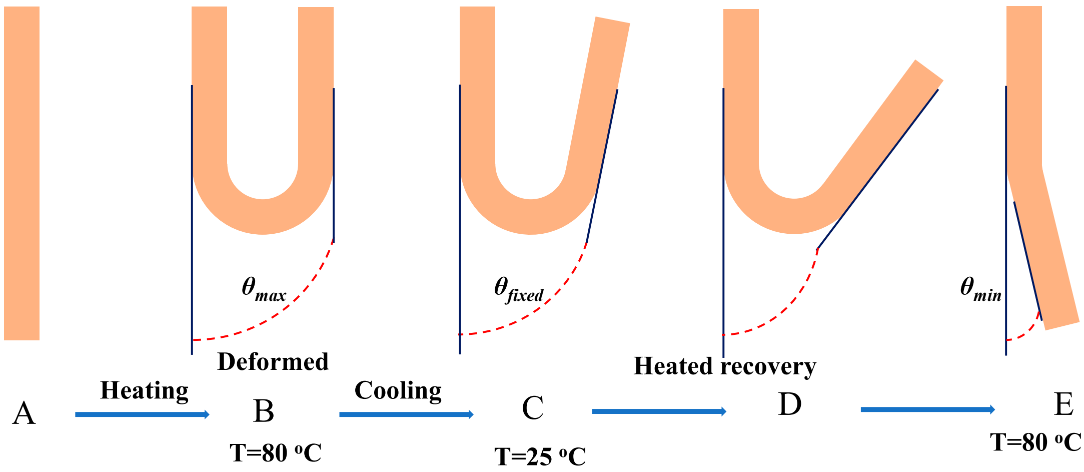


Figure S4. Schematic diagram of the shape memory performance test

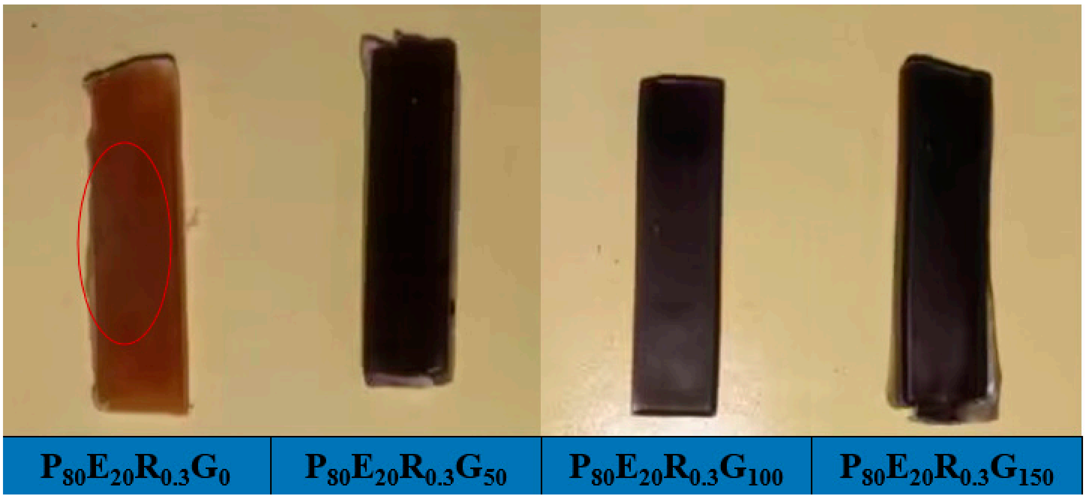


Figure S5. Pictures for anti-electrostatic test of $P_{80}E_{20}R_{0.3}G_{0}$, $P_{80}E_{20}R_{0.3}G_{50}$, $P_{80}E_{20}R_{0.3}G_{100}$ and $P_{80}E_{20}R_{0.3}G_{150}$ samples stored after one week in the ambient environment
